# Associations between Adverse Childhood Experiences (ACEs) and Prenatal Mental Health and Substance Use

**DOI:** 10.3390/ijerph20136289

**Published:** 2023-07-04

**Authors:** Tara R. Foti, Carey Watson, Sara R. Adams, Normelena Rios, Mary Staunton, Julia Wei, Stacy A. Sterling, Kathryn K. Ridout, Kelly C. Young-Wolff

**Affiliations:** 1 College of Public Health, University of South Florida, Tampa, FL 33612, USA; 2 Obstetrics and Gynecology, Kaiser Permanente, Antioch Medical Center, Antioch, CA 94531, USA; 3 Division of Research, Kaiser Permanente Northern California, Oakland, CA 94612, USAkelly.c.young-wolff@kp.org (K.C.Y.-W.); 4 Obstetrics and Gynecology, Kaiser Permanente, Dublin Medical Center, Dublin, CA 94568, USA; 5 Department of Psychiatry, Kaiser Permanente, Walnut Creek Medical Center, Walnut Creek, CA 94596, USA; 6 Department of Psychiatry and Behavioral Sciences, University of California San Francisco, San Francisco, CA 94143, USA; 7 Department of Psychiatry, Kaiser Permanente, Santa Rosa Medical Center, Santa Rosa, CA 95403, USA

**Keywords:** pregnancy, perinatal health, mental health, substance use, adverse childhood experiences, resilience, screening

## Abstract

Adverse childhood experiences (ACEs) are common and increase the risk of poor health outcomes. Resilience may offer protection against the impacts of ACEs. This study examined the association between maternal ACEs and mental/behavioral health outcomes during pregnancy overall and by resilience. The sample comprised pregnant patients in two pilot studies screened for eight ACEs and resilience during standard prenatal care in Kaiser Permanente Northern California from 1 March 2016 to 30 July 2016 (Study 1, medical centers A, B) and from 1 April 2018 to 31 March 2019 (Study 2, medical centers A, C). Early pregnancy outcomes included anxiety and depressive disorders, depression symptoms, intimate partner violence (IPV), and substance use. Multivariable logistic regression was used in this cross-sectional study to examine associations between maternal ACEs (0, 1–2, ≥3) and mental/behavioral health outcomes overall and among those with low and high resilience. Patients (n = 1084) averaged 30.8 years (SD 5.1); 41.7% were non-Hispanic White; 41.7% experienced ≥1 ACE, and 40.3% had low resilience. Patients with 1–2 ACEs or ≥3 ACEs (versus 0 ACEs) had higher odds of anxiety and depressive disorders, depressive symptoms, IPV, and any prenatal substance use (OR 1.44–4.40, *p* < 0.05). Each individual ACE was associated with ≥2 mental/behavioral health outcomes. In stratified analyses, having ≥1 ACE (vs. 0) was associated with a greater number of mental/behavioral health outcomes among patients with low versus high resilience. ACEs were associated with prenatal mental/behavioral health conditions, particularly in the context of low resilience, highlighting the importance of trauma-informed prenatal care and the need to study resilience-building interventions during pregnancy.

## 1. Introduction

Adverse childhood experiences (ACEs), or exposure to abuse, neglect, or household dysfunction prior to adulthood [1], are common: recent nationally representative surveys have estimated that 61% of adults have at least one ACE, and 25% have three or more ACEs [2]. ACEs are strongly associated with adverse medical and behavioral health outcomes in a dose–response fashion throughout the lifespan and intergenerationally [3,4,5,6,7,8]. ACEs have substantial economic costs due to factors such as chronic health problems, impaired educational achievement, reduced income and/or earning potential, and increased healthcare utilization [9,10].

Mental and behavioral health conditions, including depression, anxiety, exposure to violence/abuse, and substance use, are especially important to consider during pregnancy, as they can increase the risk of poor perinatal [11,12,13,14,15,16,17,18] and childhood outcomes [19,20,21,22,23,24]. Studies that have examined ACEs and mental health conditions in pregnancy have found associations between ACEs and prenatal depression/depressive symptoms [25,26,27,28,29,30], anxiety/anxiety symptoms [26,29], post-traumatic stress/post-traumatic stress disorder [25,26,29], and poor mental health [31]. Studies have also found an association between maternal ACEs and prenatal use of alcohol [26,27,32,33,34], tobacco or cannabis [26,27,31,32,35], and substance use or illicit drug use [26,27,31,32]. Fewer studies have examined the association between ACEs and intimate partner violence (IPV) during pregnancy. We previously found an association between ACEs and IPV [29], and Leeners et al. (2013) [35] found an association between child sexual abuse and physical, sexual, and emotional abuse in pregnancy. 

The existing literature on ACEs and mental and behavioral health outcomes has been limited by small sample sizes, participants from a limited geographic area (e.g., within one city or patients at one hospital), or focusing only on one type (e.g., sexual abuse). Additionally, studies often collapsed maternal ACEs into categories without examining the associations between individual ACEs and maternal outcomes. Research that examines associations between individual ACEs and prenatal mental/behavioral health conditions is needed to better understand the utility of documenting specific ACEs in prenatal health care settings. 

A growing body of literature has identified resilience, defined as the ability to adapt to and cope with stress and adversity [36,37], as a significant moderator of the health risks related to ACEs in the general population [38,39,40,41]. Recent studies have contributed to evidence of resilience as a psychological variable that is both a state and a trait [42,43]. It is critical to better understand the impact of resilience on the association between ACEs and perinatal health outcomes, as this can inform screening programs in clinical settings to identify patients in the highest-risk categories. Subsequently, interventions can be catered to address underlying psychological traits following childhood ACEs. Recent interventions have been developed for low-resilience patient populations [44], which could be modified for use in prenatal care settings. 

Initial resilience research, generally including studies with small sample sizes, suggests that resilience may moderate associations between ACEs and prenatal and postpartum mental/behavioral health conditions [26,29,45]. In our prior study, we found that ACEs were associated with both mental and behavioral health conditions among pregnant patients, with the strongest associations among those with low levels of current resilience [29]. However, due to the small sample size, we were unable to examine the relationship between individual ACEs and mental/behavioral health outcomes or disaggregate the use of different substances during pregnancy. Additional studies with larger sample sizes are needed to better understand whether the associations between ACEs and key mental/behavioral health outcomes during pregnancy are different for those with high versus low resilience.

The current study builds on our previously published work by combining data from two pilot sites that tested the implementation of routine ACEs and resilience screening in obstetric care. The primary objective of the study was to: (1) examine whether the number of ACEs were associated with mental and behavioral health conditions during pregnancy, and (2) to conduct stratified analyses to examine associations between ACEs and mental and behavioral health conditions separately for pregnant patients with low versus high levels of resilience. A secondary objective of this study was to examine the associations between individual ACEs and mental and behavioral health conditions in pregnancy. We used the STROBE checklist for reporting this cross-sectional study.

## 2. Materials and Methods

### 2.1. Study Site

Kaiser Permanente Northern California (KPNC) is a nonprofit, multi-specialty healthcare delivery system with over 4.3 million members and approximately 45,000 live births annually across 21 hospitals. This study combines data from two KPNC pilot studies in three medical centers that screened English-speaking pregnant patients aged ≥18 for ACEs during standard prenatal care at their second or third prenatal visit (typically between 14–23 weeks gestation) from 1 March 2016 to 30 June 2016 (Study 1, medical centers A and B) and from 1 April 2018 to 31 March 2019 (Study 2, medical centers A and C). Medical assistants provided the ACEs screening questionnaire to patients to complete in the waiting room or exam room while waiting for their physician. Physicians reviewed the questionnaire responses with patients and provided an educational handout with relevant community and educational resources and, as needed, referrals for behavioral health services. Additional information about study methods has been published previously [29,46,47,48,49]. This study received approval from the KPNC Institutional Review Board with a waiver of informed consent.

### 2.2. Participants

The study sample comprised data from 1164 pregnancies in English-speaking patients (age >18) who completed the ACEs questionnaire during standard prenatal care in either Study 1 (N = 355) or Study 2 (N = 809). Fifty-eight pregnancies were excluded for incomplete data on ACEs (n = 8), prenatal alcohol screening (n = 29), median household income (n = 4), or depression diagnosis (n = 21). For the 18 patients with >1 pregnancy that met the inclusion criteria, only data from the latest pregnancy were retained (n = 18 pregnancies excluded). The final sample included 1084 patients.

### 2.3. Measures

We assessed maternal ACEs using a modified version of the Behavioral Risk Factor Surveillance System Questionnaire [50] adapted to be appropriate for pregnant patients and easy to self-administer in a health care setting [29,46]. Patients responded “yes” or “no” to questions about whether 8 specific ACEs occurred prior to their 18th birthday. The questions were: (1) “Did you lose a parent through divorce, abandonment, death, or other reason?”, (2) “Did a parent or adult in your home ever swear at you, insult you, or put you down?”, (3) “Not including spanking, did a parent or adult in your home ever hit, beat, kick, or physically hurt you in any way?”, (4) “Did you experience unwanted sexual contact (such as fondling, or oral/anal/vaginal intercourse/penetration)?”, (5) “Did you live with anyone who had a problem with drinking or using drugs, including prescription medications?”, (6) “Did you live with anyone who was depressed, mentally ill, or attempted suicide?”, (7) “Did you live with anyone who went to jail or prison?”, (8) “Did your parents or adults in your home ever hit, punch, beat, or threaten to harm each other?”. Total possible ACEs counts ranged from 0 to 8. 

Mental and behavioral health conditions during pregnancy were extracted from the electronic health record (EHR). Depression and anxiety disorder diagnoses during pregnancy were identified using the International Statistical Classification of Diseases and Related Health Problems, Ninth Revision (ICD-9), and Tenth Revision (ICD-10) codes [29]. Depression symptoms were identified by the Patient Health Questionnaire-9 (PHQ-9), which is given to pregnant patients during standard prenatal care (depression is defined as a score >10) [51]. Intimate partner violence (IPV) was ascertained by ICD-9 and ICD-10 codes recorded in the EHR during the year before pregnancy or during pregnancy. 

Any use of alcohol, nicotine, or cannabis during early pregnancy (including prior to pregnancy recognition) was based on universal screening via a self-reported questionnaire at the entry to prenatal care (at ~8 weeks gestation). Use of nicotine was additionally based on routine screening for patient-reported current tobacco smoking at the time of the ACEs screening; patients who self-reported nicotine use on the self-administered screening questionnaire and/or self-reported current tobacco smoking at the time of the ACEs screening were coded as yes for nicotine use. Prenatal cannabis use was additionally based on a positive urine toxicology test universally given to patients at the entrance to prenatal care, to which patients consent. Patients who self-reported any cannabis use during early pregnancy and/or had a positive urine toxicology test were coded as yes for cannabis use. Any prenatal substance use during early pregnancy was defined as being positive for prenatal alcohol, nicotine, and/or cannabis use. Prenatal nicotine use alone was not included as one of our outcomes due to low prevalence (<3%). 

Patients were screened for resilience at the same time as ACEs screening using the 10-item Connor-Davidson Resilience Scale (CD-RISC 10) [52]. This questionnaire is a validated, self-reported measure of past-month resilience that has previously been used in research with pregnant and postpartum patients [29,52,53]. Questions address components of psychological resilience, such as the ability to bounce back after hardship, handle unpleasant or painful feelings, and adapt to change. Answer options are scored from 0 (“not at all true”) to 4 (“true nearly all the time”), with total scores ranging from 0 to 40 [45,54]. Scores were dichotomized into low (<32) and high resilience (>32) based on the national average [52], as conducted by our team previously [29].

Socio-demographics were obtained from the EHR and included the patient’s age at ACEs screening, race/ethnicity (Asian/Pacific Islander, Black, Hispanic, non-Hispanic White, other/unknown), and parity. Neighborhood median household income was based on census tract data and was divided into terciles: $0–$82,999, $83,000 to $105,999, and $106,000 or higher. 

### 2.4. Statistical Analysis

Frequencies and percentages were used to describe socio-demographics (age, race/ethnicity, neighborhood median household income), clinical characteristics (parity, resilience), ACE count (0, 1–2, and ≥3), and individual ACEs. ACE count categories were consistent with what we have previously published [29] and provided clinically meaningful information about ACEs (i.e., none, some, and many). While >4 ACEs are generally considered to represent high-risk for adverse outcomes [6], only a small proportion of patients in our sample reported >4 ACEs, and, thus, our highest category is >3 ACEs. Seven multivariable logistic regression models were used to compare the odds of each of the mental/behavioral health conditions by ACE count and by individual ACEs and to compare the odds of mental/behavioral health conditions of interest by ACE count, stratified by patients with high versus low resilience [55,56]. We stratified by high/low resilience, as conducted in our prior work, due to our limited power to test for statistical significance of interaction and because stratification would provide clinically meaningful data to our clinicians and healthcare system to understand whether the combination of ACEs and low resilience is associated with highest odds of behavioral and mental health outcomes. All regression analyses were adjusted for maternal age categories, race/ethnicity, parity, and median neighborhood income categories based on previous literature and the availability of variables from the pilot study data and electronic medical records. To determine statistical significance, we first considered two-sided *p*-values of <0.05 statistically significant. Additionally, we applied the Benjamini-Hochberg procedure to decrease the potential of false positive results [57]. In brief, for this correction, we ranked the *p*-values of each of the seven mental and behavioral health outcomes for each category of ACE predictors (1–2 and >3 ACEs). We calculated the Benjamini-Hochberg critical value for each p-value using the formula ([i/m]*Q), where I = rank of *p*-value [ranging from 1 to 7], m = the total number of tests [7] and Q = the false discover rate [0.05]. Statistical analyses were performed in SAS 9.4. Odds ratio results are presented by effect size, with small effect >1.22, medium effect >1.86, and large effect >3.00 [58].

## 3. Results

The sample of pregnant patients (n = 1084) was primarily non-Hispanic White (41.7%), averaging 30.8 years (SD 5.1) (Table 1). Participants reported a mean of 1.0 ACE (SD 1.6); 58.2% reported 0 ACEs, 27.2% reported 1–2 ACEs, and 14.6% reported ≥3 ACEs. The most commonly reported ACEs were loss of a parent (23.4%), emotional abuse (15.6%), having lived with someone with a substance use problem (15.5%), and having lived with someone depressed, mentally ill, or suicidal (14.2%). Fewer patients had low resilience (40.3%) compared to high resilience (59.7%) based on CD-RISC scores (Table 2). 

In multivariable logistic regression models, having ≥3 vs. 0 ACEs was significantly associated with higher odds of all mental and behavioral health outcomes during pregnancy, with the exception of prenatal alcohol use (Table 3 and Table 4). The strength of the associations for ≥3 vs. 0 ACEs ranged from small for any substance use during early pregnancy to large for IPV. Further, having 1–2 vs. 0 ACEs was significantly associated with higher odds of mental and behavioral health conditions except for the use of substance use during early pregnancy (including alcohol, cannabis, and any substance use). The strength of the associations ranged from small for depressive disorder and depression symptoms to medium for IPV (Table 3 and Table 4). Upon applying the Benjamini-Hochberg procedure, only anxiety fell out of significance for having 1–2 ACEs.

For the secondary objective, all individual ACEs were significantly associated with depressive disorder, and all except for living with someone who went to jail or prison were significantly associated with IPV. Childhood emotional abuse was associated with the greatest number of mental/behavioral health outcomes (6 outcomes), followed by sexual abuse (5 outcomes), and living with someone who was depressed, mentally ill, or suicidal (4 outcomes) (Table 3 and Table 4). 

**Figure 1:**
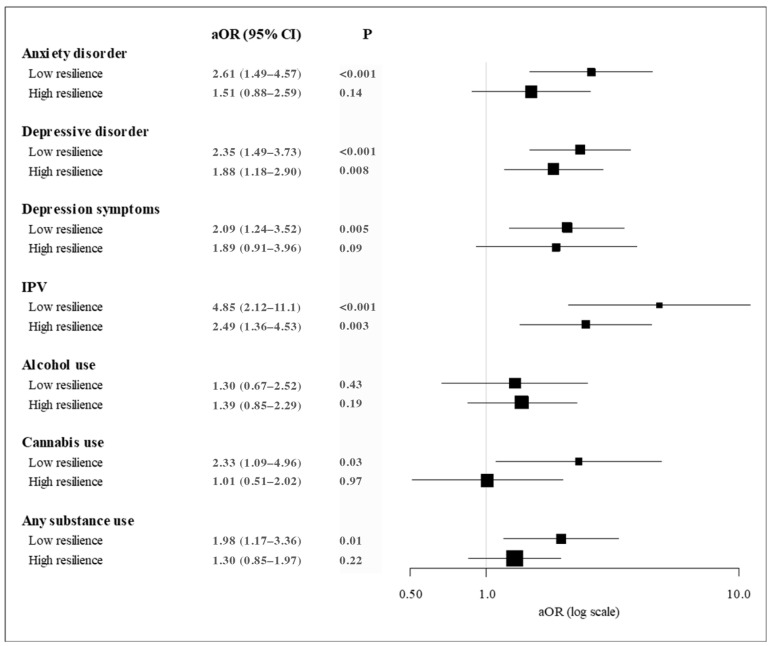
Adjusted Odds Ratios of Prenatal Mental and Behavioral Health Conditions by Exposure to One or More Adverse Childhood Experiences (ACEs) Stratified by Low (N = 429) and High (N = 636) Resilience; Notes. aOR = adjusted odds ratio. ACE = Adverse childhood experience. IPV = Intimate partner violence diagnosis. CI = confidence interval. 1+ vs. 0 ACEs for each outcome/category. Resilience was missing for 19 patients. Adjusted for age, race/ethnicity, median neighborhood household income, and parity. For only the prenatal cannabis use outcome, the Asian race was combined with the multiple/other/unknown race category so the model would converge. Any substance use was defined as being positive for any alcohol, nicotine, and/or cannabis use during early pregnancy.

In multivariable models of outcomes stratified by low/high resilience, we dichotomized exposure to ACEs (1+ vs. 0 ACEs) due to the smaller sample sizes of patients with low (n = 429) and high resilience (n = 636). Among patients with low resilience, having 1+ ACE was associated with increased odds of 6 mental/behavioral health outcomes during pregnancy: anxiety disorder (medium effect), depressive disorder (medium effect), depressive symptoms (medium effect), IPV (large effect), cannabis use during early pregnancy (medium effect), and any substance use during early pregnancy (medium effect) (Figure 1). In contrast, among patients with high resilience, having 1+ ACE was significantly associated with only two mental/behavioral health outcomes during pregnancy: depressive disorder (medium effect) and IPV (medium effect).

## 4. Discussion

This study sought to: (1) examine whether the number of ACEs were associated with mental and behavioral health conditions during pregnancy, and (2) to conduct stratified analyses to examine associations between ACEs and mental and behavioral health conditions separately for pregnant patients with low versus high levels of resilience. Secondarily, we examined the associations between individual ACEs and mental and behavioral health conditions in pregnancy. Results from our diverse sample of pregnant patients screened for ACEs during standard prenatal care found evidence of a relationship between the number of ACEs and mental/behavioral health conditions in pregnancy, with the strongest relationships for the greatest category of ACEs, consistent with previous studies [26,29,34,59]. Compared to pregnant patients without ACEs, those with 1–2 ACEs had 1.5 to 2.5 times the odds of having an anxiety or depressive disorder, depression symptoms, IPV, or any substance use during early pregnancy, and those with ≥3 ACEs had 1.8 to 4.7 times the odds of having an anxiety or depressive disorder, depression symptoms, IPV, or any substance use during early pregnancy. Moreover, similar to our prior work [26,29,45], the strongest associations were between ACEs and IPV. Notably, many of the associations between the number of ACEs and mental/behavioral health conditions were stronger for pregnant patients with low versus high resilience. Finally, each individual ACE was associated with at least two prenatal mental/behavioral health conditions. 

Results add support to recent efforts to assess maternal ACEs during pregnancy [46,48,60]. While a growing number of prenatal and obstetric healthcare settings are implementing ACEs screening, it remains unclear how best to record or report maternal ACEs. Many studies utilize ACE counts, categorize ACEs into none/low/high categories, or focus on one specific type of ACE (e.g., childhood sexual abuse) [27,32,33,34,59,61,62,63], while others aggregate certain ACEs into subtypes, such as “dysfunction” and “neglect” [31], “maltreatment” [25,26], or “violence” [27]. Findings from this study suggest that specific ACEs, including experiencing emotional abuse and having a parent in jail or prison, are particularly important predictors of mental/behavioral health conditions in pregnancy. Future studies are needed to continue to identify best practices for screening and recording maternal ACEs [64], and clinical perinatal settings may consider recording both the overall number of maternal ACEs, alongside individual ACE responses. 

Resilience is an important consideration in prenatal care settings. This study adds to a small but growing body of literature demonstrating the impact of resilience on the relationship between ACEs and prenatal mental/behavioral health conditions [29]. There is evidence supporting resilience as a mixed state-trait psychological variable [65,66], suggesting that resilience-building interventions, particularly when tailored to an individual’s underlying psychological traits [67], may be an effective intervention strategy after ACE exposure. While efforts have been made to implement programs to improve patient resilience through strategies such as mindfulness and psychosocial skills training [68] or trauma-informed care during and after ACE screening [39], such interventions are limited in prenatal settings, and it remains unknown the extent to which maternal improvements in resilience may impact risk behaviors and outcomes for mothers and babies.

Nearly half of the patients in this pilot study experienced ACEs, highlighting the importance of implementing trauma-informed care in obstetric settings. Trauma-informed care involves the recognition of trauma on health and having staff employ practices to actively avoid re-traumatization of patients in order to promote healing [69]. One element of trauma-informed involves screening for ACEs during prenatal care [27,70]. Based on patient feedback, this screening should be conducted with provider empathy [64] and in a private exam room [60] by either a physician or midwife [60] or behavioral health counselor [64]. On the other hand, some clinical settings may opt for implementing universal trauma-informed care under the assumption that any patient may have experienced childhood or other neglect and/or abuse. While this approach may be particularly beneficial in limited-resource settings, we believe that this study demonstrates value in ACE and resilience screening and that patients with such positive screens may benefit from additional screening (e.g., substance use), referrals (e.g., mental health), and interventions (e.g., therapy, resilience-building).

### Limitations

Pilot studies took place within three KPNC medical centers with English-speaking adult patients who were screened for ACEs at the start of their second trimester, and results may not be generalizable to non-English speaking, adolescent, or uninsured patient populations. As this study was conducted as a part of standard prenatal care, certain demographic variables that may be of interest were not available for use in statistical models (e.g., maternal education, marital status, employment). However, some of these variables may be on the causal pathway and may not be appropriate to include in analyses. Additionally, ACEs screening data did not include information about severity, duration, or age at exposure, neglect, or other important factors such as systematic racism, and additional studies are needed to examine the impact of these important variables on prenatal mental and behavioral health. Because of the distribution of ACE counts within our study population, our highest category of ACEs was >3, representing 14.6% of the participants. As many of our mental and behavioral outcomes were relatively rare, some of our non-significant findings may be due to Type 2 errors. The number of patients with certain ACEs was sometimes small when broken out by mental/behavioral health outcomes, which limited statistical power to detect differences in outcomes by individual ACEs. We were limited to substance use during early pregnancy and were unable to distinguish between substance use that occurred prior to versus after pregnancy recognition. Patients who endorsed any alcohol use during early pregnancy may have been reporting infrequent use that occurred only prior to pregnancy recognition; this may explain the lack of association between ACEs and prenatal alcohol use. Likewise, we were unable to include federally illicit substance use due to low rates of patient reports (<1%) and, therefore, are unable to address the relationships between ACEs and perinatal opioid, stimulant, and other illicit substance use. Additional studies that include data on continued substance use after pregnancy recognition are needed. 

## 5. Conclusions

Using pilot data from an integrated healthcare delivery system with screening for ACEs during standard prenatal care in a diverse sample of pregnant patients, this study found that both count and individual ACEs were associated with mental and behavioral health conditions during pregnancy. Further, findings suggest that high resilience may buffer some of the deleterious effects of ACEs. Future research should focus on how resilience-based strategies can buffer the impact of ACEs on health outcomes. Our results suggest that clinicians and policymakers should support resilience-building clinical environments by supporting training and practice of trauma-informed care. Examples of action steps toward a trauma-informed care environment can be found through organizations, including The Substance Abuse and Mental Health Services Administration and ACEs Aware [71]. Results highlight the importance of trauma-informed prenatal care and underscore the need for future studies that investigate the efficacy of resilience-building interventions during the prenatal period.

## Figures and Tables

**Table 1:** Patient Demographics (N = 1084).

	N (%)
Age in years, categories	
18–28	353 (32.6)
29–33	400 (36.9)
34–47	331 (30.5)
Age in years, mean (SD)	30.8 (5.1)
Race/ethnicity	
Non-Hispanic White	452 (41.7)
Hispanic	260 (24.0)
Asian/Pacific Islander	198 (18.3)
Black	126 (11.6)
Multiple, other, or unknown	48 (4.4)
Parity	
Nulliparous	407 (37.6)
Primiparous	406 (37.5)
Multiparous	271 (24.0)
ACE count, categories	
0	631 (58.2)
1	202 (18.6)
2	93 (8.6)
3–8	158 (14.6)
ACE count, mean (SD)	1.0 (1.6)

Notes. ACE = Adverse childhood experience. SD = Standard deviation.

**Table 2:** Adverse Childhood Experiences (ACEs) Exposure and Prenatal Mental and Behavioral Health Conditions.

	Overall N (Col %)	Prenatal Mental and Behavioral Health Conditions, N (Row %)						
		Anxiety Disorder	Depressive Disorder	Depression Symptoms	IPV Diagnosis	Alcohol Use During Early Pregnancy	Cannabis Use During Early Pregnancy	Any Substance Use During Early Pregnancy
Total	1084	133 (12.3)	210 (19.4)	112 (10.3)	92 (8.5)	117 (10.8)	84 (7.7)	201 (18.5)
Number of ACEs								
0	631 (58.2)	56 (8.9)	88 (13.9)	46 (7.3)	29 (4.6)	59 (9.4)	37 (5.9)	94 (14.9)
1–2	295 (27.2)	40 (13.6)	69 (23.4)	36 (12.2)	32 (10.8)	40 (13.6)	26 (8.8)	65 (22.0)
≥3	158 (14.6)	37 (23.4)	53 (33.5)	30 (19.0)	31 (19.6)	18 (11.4)	21 (13.3)	42 (26.6)
Resilience *								
Low (≤32)	429 (40.3)	70 (16.3)	113 (26.3)	78 (18.2)	38 (8.9)	43 (10.0)	39 (9.1)	81 (18.9)
High (>32)	636 (59.7)	62 (9.7)	95 (14.9)	33 (5.2)	52 (8.2)	74 (11.6)	42 (6.6)	117 (18.4)
Individual ACEs								
Loss of parent								
Yes	254 (23.4)	39 (15.4)	68 (26.8)	38 (15.0)	36 (14.2)	37 (14.6)	23 (9.1)	61 (24.0)
No	830 (76.6)	94 (11.3)	142 (17.1)	74 (8.9)	56 (6.7)	80 (9.6)	61 (7.3)	140 (16.9)
Emotional abuse								
Yes	169 (15.6)	37 (21.9)	52 (30.8)	35 (20.7)	29 (17.2)	19 (11.2)	21 (12.4)	43 (25.4)
No	915 (84.4)	96 (10.5)	158 (17.3)	77 (8.4)	63 (6.9)	98 (10.7)	63 (6.9)	158 (17.3)
Physical abuse								
Yes	67 (6.2)	13 (19.4)	21 (31.3)	--	11 (16.4)	--	--	16 (23.9)
No	1017 (93.8)	120 (11.8)	189 (18.6)	--	81 (8.0)	--	--	185 (18.2)
Sexual abuse								
Yes	79 (7.3)	16 (20.3)	27 (34.2)	13 (16.5)	17 (21.5)	14 (17.7)	15 (19.0)	29 (36.7)
No	1005 (92.7)	117 (11.6)	183 (18.2)	99 (9.9)	75 (7.5)	103 (10.2)	69 (6.9)	172 (17.1)
Lived with someone:								
-With substance use problem								
Yes	168 (15.5)	36 (21.4)	54 (32.1)	24 (14.3)	30 (17.9)	17 (10.1)	20 (11.9)	40 (23.8)
No	916 (84.5)	97 (10.6)	156 (17.0)	88 (9.6)	62 (6.8)	100 (10.9)	64 (7.0)	161 (17.6)
-Who was depressed, mentally ill, or suicidal								
Yes	154 (14.2)	36 (23.4)	54 (35.1)	33 (21.4)	27 (17.5)	14 (9.1)	15 (9.7)	31 (20.1)
No	930 (85.8)	97 (10.4)	156 (16.8)	79 (8.5)	65 (7.0)	103 (11.1)	69 (7.4)	170 (18.3)
-Who went to jail or prison								
Yes	87 (8.0)	18 (20.7)	26 (29.9)	12 (13.8)	12 (13.8)	--	17 (19.5)	27 (31.0)
No	997 (92.0)	115 (11.5)	184 (18.5)	100 (10.0)	80 (8.0)	--	67 (6.7)	174 (17.5)
-Who hit, punched, beat, or threatened to harm another adult in the home								
Yes	91 (8.4)	15 (16.5)	24 (26.4)	15 (16.5)	18 (19.8)	10 (11.0)	15 (16.5)	24 (26.4)
No	993 (91.6)	118 (11.9)	186 (18.7)	97 (9.8)	74 (7.5)	107 (10.8)	69 (6.9)	177 (17.8)

Notes. ACE = Adverse childhood experience. IPV = Intimate partner violence diagnosis. Percentages may not add up to 100 due to rounding. Patients could have more than one prenatal mental or behavioral health condition. * Resilience was missing for 19 patients. Any substance use during early pregnancy was defined as being positive for alcohol, nicotine, and/or cannabis use during early pregnancy. Cells replaced with ‘--’ indicate cell counts of less than 10 patients or cell counts that could be used to derive cell counts with less than 10 patients; these cells were suppressed to protect patient identity.

**Table 3:** Adjusted Odds Ratios of Prenatal Mental Health Conditions by Adverse Childhood Experiences (ACEs) Count and Individual ACE (N = 1084).

	Anxiety Disorder		Depressive Disorder		Depression Symptoms		IPV Diagnosis	
	aOR (95% CI)	*p*	aOR (95% CI)	*p*	aOR (95% CI)	*p*	aOR (95% CI)	*p*
**ACE count**								
1–2 vs. 0 ACEs	1.56 (1.01–2.42)	0.045	1.83 (1.28–2.61)	<0.001 *	1.73 (1.08–2.77)	0.02 *	2.48 (1.46–4.22)	<0.001 *
≥3 vs. 0 ACEs	2.90 (1.81–4.66)	<0.001 *	2.83 (1.88–4.27)	<0.001 *	3.00 (1.78–5.03)	<0.001 *	4.40 (2.52–7.67)	<0.001 *
**Individual ACEs**								
Loss of parent	1.33 (0.88–2.00)	0.18	1.69 (1.20–2.37)	0.003	1.71 (1.11–2.63)	0.02	2.01 (1.27–3.17)	0.003
Emotional abuse	2.31 (1.50–3.56)	<0.001	2.01 (1.38–2.92)	<0.001	3.06 (1.94–4.81)	<0.001	2.61 (1.60–4.25)	<0.001
Physical abuse	1.79 (0.93–3.43)	0.08	1.95 (1.12–3.39)	0.02	1.46 (0.69–3.08)	0.33	2.13 (1.05–4.34)	0.04
Sexual abuse	1.91 (1.05–3.48)	0.03	2.38 (1.44–3.95)	<0.001	1.62 (0.85–3.08)	0.14	3.08 (1.68–5.67)	<0.001
*Lived with someone:*								
-With substance use problem	2.07 (1.34–3.21)	0.001	2.03 (1.39–2.96)	<0.001	1.60 (0.96–2.64)	0.07	2.83 (1.73–4.63)	<0.001
-Who was depressed, mentally ill, or suicidal	2.32 (1.50–3.61)	<0.001	2.38 (1.62–3.48)	<0.001	3.36 (2.09–5.40)	<0.001	2.76 (1.67–4.57)	<0.001
-Who went to jail or prison	1.97 (1.11–3.49)	0.02	1.84 (1.11–3.03)	0.02	1.17 (0.60–2.27)	0.65	1.50 (0.77–2.93)	0.24
-Who hit, punched, beat, or threatened to harm another adult in the home	1.41 (0.77–2.55)	0.26	1.46 (0.88–2.41)	0.14	1.84 (1.00–3.39)	0.49	3.04 (1.69–5.46)	<0.001

Notes. aOR = Adjusted odds ratio. ACE = Adverse childhood experience. IPV = Intimate partner violence diagnosis. All models were adjusted for age, race/ethnicity, median neighborhood household income, and parity. * indicates statistical significance after applying the Benjamini-Hochberg adjustment for false discovery rates.

**Table 4:** Adjusted Odds Ratios of Prenatal Behavioral Health Conditions by Adverse Childhood Experiences (ACEs) Count and Individual ACE (N = 1084).

	Alcohol Use During Early Pregnancy		Cannabis Use During Early Pregnancy		Any Substance Use During Early Pregnancy	
	aOR (95% CI)	*p*	aOR (95% CI)	*p*	aOR (95% CI)	*p*
**ACE count**						
1–2 vs. 0 ACEs	1.43 (0.93–2.21)	0.11	1.28 (0.73–2.25)	0.38	1.44 (1.00–2.07)	0.053
≥3 vs. 0 ACEs	1.13 (0.64–2.01)	0.67	1.90 (1.03–3.51)	0.039 *	1.72 (1.12–2.65)	0.01 *
**Individual ACEs**						
Loss of parent	1.49 (0.97–2.28)	0.07	0.88 (0.51–1.52)	0.65	1.29 (0.90–1.84)	0.16
Emotional abuse	1.04 (0.61–1.76)	0.89	1.93 (1.09–3.43)	0.02	1.62 (1.08–2.42)	0.02
Physical abuse	0.98 (0.43–2.24)	0.97	1.27 (0.49–3.26)	0.62	1.51 (0.82–2.79)	0.18
Sexual abuse	1.81 (0.96–3.39)	0.07	2.74 (1.40–5.36)	0.003	2.60 (1.56–4.34)	<0.001
*Lived with someone:*						
-With substance use problem	0.83 (0.48–1.45)	0.52	1.64 (0.92–2.91)	0.09	1.29 (0.85–1.94)	0.23
-Who was depressed, mentally ill, or suicidal	0.72 (0.40–1.31)	0.28	1.36 (0.72–2.57)	0.35	1.04 (0.67–1.62)	0.87
-Who went to jail or prison	0.54 (0.23–1.28)	0.16	2.13 (1.11–4.05)	0.02	1.65 (0.99–2.74)	0.06
-Who hit, punched, beat, or threatened to harm another adult in the home	0.94 (0.47–1.89)	0.86	2.54 (1.29–4.97)	0.007	1.52 (0.91–2.55)	0.11

Notes. aOR = Adjusted odds ratio. ACE = Adverse childhood experience. Adjusted for age, race/ethnicity, median neighborhood household income, and parity. For only the prenatal cannabis use outcome, the Asian race was combined with the multiple/other/unknown race category so the model would converge. Any substance use during early pregnancy was defined as being positive for alcohol, nicotine, and/or cannabis use during early pregnancy. * indicates statistical significance after applying the Benjamini-Hochberg adjustment for false discovery rates.

## Data Availability

The data are not publicly available because authors do not have permission to share.
